# Beyond daylight: emergent cardiac catheterization in congenital heart disease during evenings and nights: clinical demand and logistical realities

**DOI:** 10.3389/fcvm.2026.1752520

**Published:** 2026-04-13

**Authors:** Nathalie Mini, Martin B. E. Schneider, Ehrenfried Schindler, Boulos Asfour, Marian Mikus

**Affiliations:** 1 Cardiac Catheterization Laboratories, German Paediatric Heart Center, University Hospital Bonn, Bonn, Germany; 2 Department of Anesthesiology and Intensive Care Medicine, University Hospital Bonn, Bonn, Germany; 3 Department of Pediatric Cardiac Surgery, German Pediatric Heart Centre, University Hospital Bonn, Bonn, Germany

**Keywords:** congenital heart disease, emergency cardiac catheterization, neonatal cardiac intervention, off-hours intervention, pediatric interventional cardiology

## Abstract

**Background:**

Emergency cardiac catheterizations (ECC) during off-hours are high risk due to limited staffing and resources. To date, no systematic data are available on the outcomes of such emergent procedures for congenital heart disease (CHD).

**Objectives:**

To the best of our knowledge, this study is among the first to systematically evaluate outcomes, procedural characteristics, and multidisciplinary involvement in off-hours ECC among patients with CHD

**Methods:**

We retrospectively analyzed all ECC performed during evenings and nights over two and half years at a single tertiary center. Data included demographics, indications, interventions, staffing, and outcomes.

**Results:**

Between January 2023 and November 2025, a total of 2,050 procedures were performed; of these, 37 (1.8%) were emergent off-hours cases. Median age was 25 days (range: 1 day–14 years); median weight was 3.9 kg (range: 2–50 kg). Indications included critical cyanosis (40.5%), cardiogenic shock (19%), postoperative instability (37.8%), and interventional complications (2.7%). Interventions included Rashkind atrioseptostomy, pulmonary/aortic balloon angioplasty, shunt, pulmonary and coarctation stenting, and postoperative rescue procedures, accounting for 89% of cases. All ECCs were performed by a single interventionalist. Nursing staff support was required in 48% of performed interventions, primarily those involving stenting and dilation. Rashkind-guided echocardiography was performed by anesthesiology (n = 7). The surgical team was on-site in 40% of cases. All patients who underwent intervention showed clinical improvement; ECMO was avoided in four cases and weaned in two. No early complications were observed. One late femoral arteriovenous fistula required surgical revision.

**Conclusions:**

Emergent off-hours catheterizations in CHD can achieve favorable outcomes when performed by a skilled multidisciplinary team. Success depends not only on physician expertise but also on institutional readiness, effective organization, and seamless team coordination.

## Introduction

Emergent cardiac catheterization is a critical intervention for managing life-threatening events in patients with congenital heart disease (CHD), a population with unique anatomical and physiological challenges (1, 2). While scheduled catheterizations are standard in CHD management, emergent interventions are unpredictable and time sensitive, particularly when complications arise outside routine hours. Nighttime and evening emergencies pose additional challenges due to limited staffing, resource availability, and potential delays in diagnosis and intervention (3). Most published reports that evaluated outcomes of off-hours emergent cardiac interventions focused on adult cardiology populations, particularly patients with acute coronary syndromes (3–5). These studies highlight the impact of institutional preparedness and team coordination on procedural success, but their findings may not be directly generalizable to the pediatric CHD population, given the distinct anatomical complexity, hemodynamic variability, and procedural risks.

To date, data on emergent off-hours catheterizations in CHD patients remain limited. This study addresses this gap by reviewing outcomes, procedural characteristics, and logistical challenges of emergent catheterizations performed during evenings and nights in CHD patients. Importantly, all procedures were conducted by a single experienced interventionalist, allowing focused evaluation of operator-dependent and institutional factors. Insights from these high-risk off-hour interventions can guide improvements in clinical protocols, team preparedness, and resource allocation to optimize safety and outcomes in this vulnerable population.

## Patients and methods

**Figure 1:**
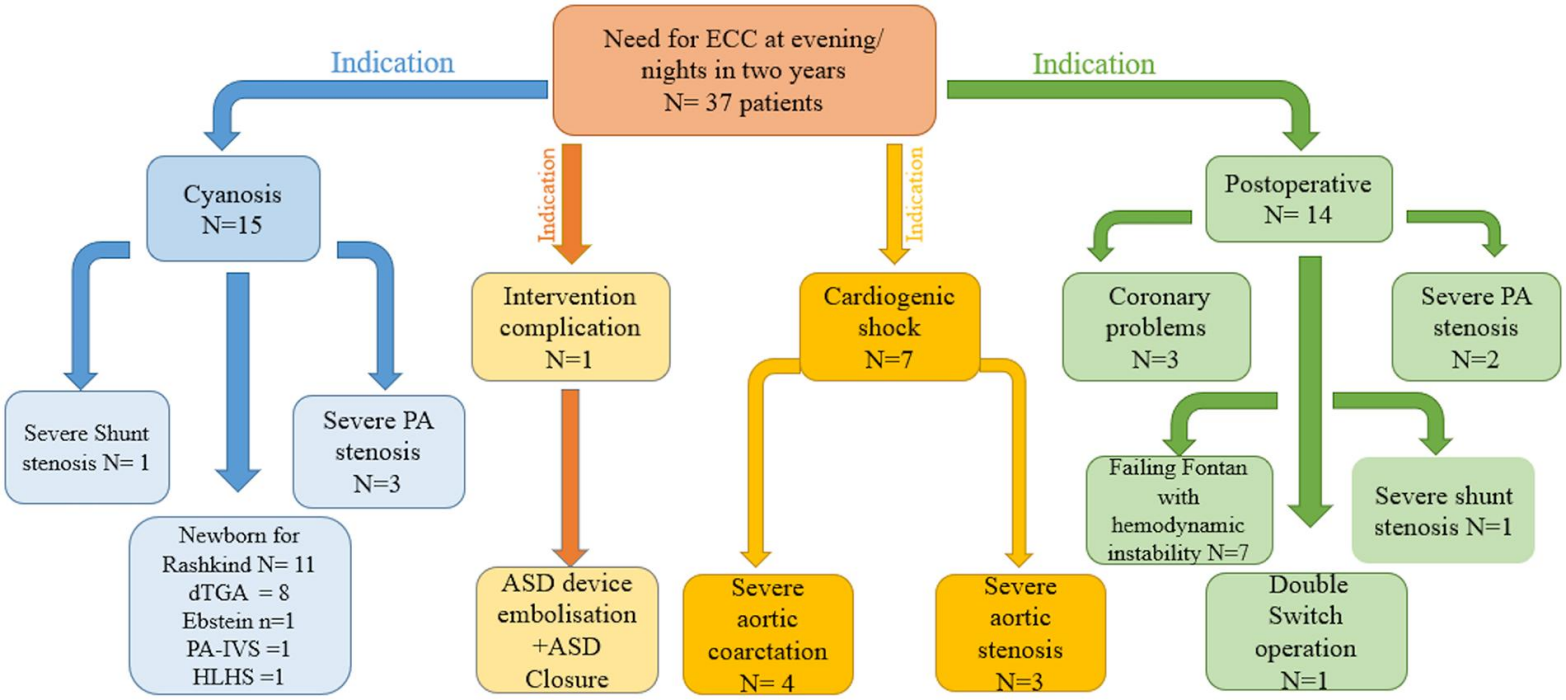
Summary of indications and outcomes of emergent Off-hours catheterization procedures. Caption 1: ECC: emergent cardiac catheterization, ASD, atrial septal defect; d-TGA, dextro-transposition of the great arteries; HLHS, hypoplastic left heart syndrome; PA, pulmonary artery; PA-IVS, pulmonary atresia with intact ventricular septum. One case of PA stenosis was intervened immediately after the operation and was therefore classified in the postoperative group.

Between January 2023 and November 2025, 2,050 cardiac catheterizations were performed at the German Paediatric Heart Center, University Hospital Bonn. Of these, 37 patients with CHD who underwent emergent catheterization during evening or nighttime hours—including weekdays and weekends—and were managed by a single interventionalist were included. Patients who required multiple interventionalists or procedures performed during regular working hours were excluded. The indications for emergency cardiac catheterization (ECC) are summarized in Figure 1.

Collected data included indication for emergent catheterization, age, weight, type of intervention, early and late complications, procedure-related mortality, need for extracorporeal membrane oxygenation (ECMO), radiation time, requirement for assistance during the procedure, and need for reoperation. Emergency Catheterizations are summarized in Table 1.

**Table 1:** Summary of emergency catheterizations.

Category	Patients (%)	Interventions performed	Outcome summary
Critical cyanosis	15 (40.5)	Rashkind, PDA stent, pulmonary/shunt intervention	All stabilized, no ECMO needed
Cardiogenic shock	7 (19)	Aortic stent/dilation	All improved LV function/cardiac output
Postoperative instability	14 (37.8)	Fenestration recanalization, PA/azygos stenting, coronary angiogram	Two avoided ECMO, two required re-surgery, one required conversative treatment, one late complication with AV fistula
Interventional complication	1 (2.7)	Device retrieval, ASD reclosure	No residual defect
			

PDA, patent ductus arteriosus; MBTS, modified Blalock–Taussig shunt; ECMO, extracorporeal membrane oxygenation; LV, left ventricle; LPA, left pulmonary artery; ASD, atrial septal defect; PA, pulmonary artery.

### Statistical analysis

Continuous variables are presented as median ± interquartile range and categorical variables as count (percentage). Analyses were performed using SPSS version 22. Histograms of radiation time and radiation dose were generated using SPSS version 20 to visualize their distributions.

### Ethical statement

This retrospective study analyzed anonymized data from emergent cardiac catheterization procedures performed as part of standard clinical care in human participants. The study was approved by the local ethics committee (reference number 2025-277-BO), which waived the requirement for informed consent.

## Results

### Patient characteristics

**Figure 2:**
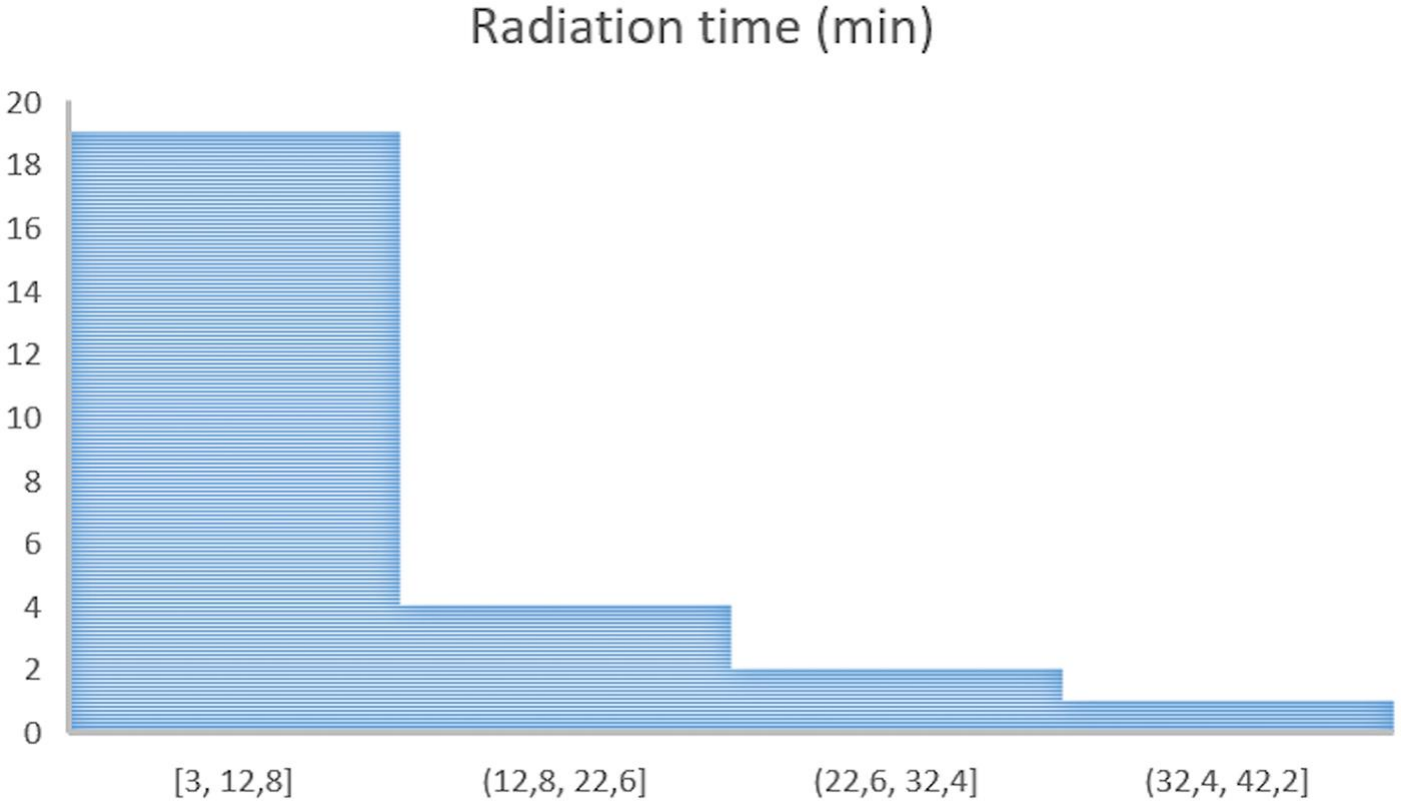
Histograms showing the distribution of radiation time (minutes) during off-hours emergent catheterizations. Caption 2: The Rashkind procedures (*n* = 11) were performed with the assistance of thoracic echocardiography and were therefore excluded from the histogram.

Over two years, 2,050 cardiac catheterizations were performed; 37 (1.8%) required emergency intervention during off-duty hours, with a single interventionalist available. Median age was 25 days (range: 1 day–14 years), median weight was 3.9 kg (range: 2–50 kg), and median radiation time was 9 min (range: 3–35 min) (Figure 2). An overview of case distribution, interventions, and outcomes is shown in Table 1.

1. Critical Cyanosis (15 patients, 40.5%)

Eleven patients underwent Rashkind procedures, most commonly for dextro-transposition of the great arteries (d-TGA; *n* = 8), with additional cases in Ebstein anomaly (*n* = 1), HLHS (*n* = 1), and PA/IVS (*n* = 1). Three patients required interventions for severe pulmonary stenosis: one with critical PS, one with PA/IVS and a PDA stent, and one post-truncus repair with thrombotic right pulmonary artery obstruction and severe left pulmonary artery stenosis. This last patient was a candidate for ECMO; the intervention was performed with both surgical backup and ECMO readiness. One additional patient required stenting of a severely stenotic MBTS. All patients stabilized without ECMO. Details by diagnosis, age, and intervention are provided in Table 2.

2. Cardiogenic Shock (7 patients, 19%)

**Table 2:** Emergency catheterization procedures by diagnosis and Age group.

Diagnosis	n	Age Range (days–years)	Intervention
d-TGA/ HLHS with restrictive ASD	9	1–10 days	Rashkind
Ebstein anomaly with restrictive ASD	1	2 days	Rashkind
PA/IVS	2	2–5 days	Rashkind+PDA stent
Critical pulmonary stenosis	1	7 days	Balloon valvuloplasty
Truncus arteriosus (post-op PA stenosis)	1	1 month	LPA/RPA stenting
MBTS stenosis	1	20 days	Shunt stenting
Severe aortic stenosis	3	5–14 days	Balloon valvuloplasty
Severe aortic coarctation	4	3–30 days	Balloon dilation/stent
Post-Fontan failure	7	3–14 years	Fenestration, LPA/azygos stent
Post-Ebstein repair (coronary injury)	1	1 year	Angio+surgical revision
Post-arterial switch with coronary stenosis	1	1 month	Coronary angio+surgical revision
Post-suboartic resection with coronary thrombosis	1	4 years	Conversative therpay
Severe PA stenosis	2	6 months	RPA/LPA stenting
Double switch with tunnel stenosis	1	10 months	Venous tunnel stent
HLHS post-Norwood with clipped shunt	1	7 days	Shunt dilation
Device embolization (ASD)	1	3 years	Device retrieval+reclosure

PA-IVS, Pulmonary atresia with intact ventricular septum; RPA, Right pulmonary artery; LPA, Left pulmonary artery; HLHS, Hypoplastic left heart syndrome; ASD, Atrial septal defect; d-TGA, Dextro-transposition of the great arteries.

Three patients with severe aortic stenosis underwent balloon valvuloplasty, and four with coarctation underwent balloon dilation (*n* = 1) or stenting (*n* = 3). All patients showed recovery of left ventricular function.

3. Postoperative Complications (14 patients, 37.8%)

Seven post-Fontan patients required emergent catheterization; five underwent catheter-based reinterventions (fenestration reopening or LPA/azygos stenting), and two required immediate surgical revision. Two patients developed coronary stenosis following Ebstein repair and an arterial switch operation. Both required ECMO support, and catheterization demonstrated coronary compression, necessitating urgent surgical correction. Another patient with a thrombophilic disorder experienced a STEMI on ECG after resection of subaortic stenosis; catheterization revealed thrombosis in the distal circumflex artery, and the patient received anticoagulation therapy. Two additional patients with severe pulmonary artery stenosis were successfully treated with PA stenting. One patient with ccTGA and tunnel stenosis required ECMO support but was successfully weaned after tunnel stenting. Another patient with HLHS and severe cyanosis underwent balloon dilation of a clipped shunt and was extubated within 48 h.

4. Catheterization-Related Complications (1 patient, 2.7%)

One patient who had an elective ASD closure experienced embolization of the occluder 36 h post-procedure, requiring emergency intervention. The device was successfully retrieved and redeployed without sequelae.

### Staff involvement and resources

Most procedures were performed by a single interventionalist, supported by nursing and anesthesia/ICU staff. Assistant physician involvement was rare. ECMO and surgical backup were mobilized for selected high-risk and postoperative cases (Table 3).

**Table 3:** Staff involvement and resource Use.

Case Category	N	MTA-Support	ECMO-Standby	Surgical Team
Rashkind procedures	11	1	2	1
Pulmonary lesions	4	3	2	2
Aortic stenosis	3	1	No	No
Aortic coarctation	4	4	No	No
Post-Fontan complications	7	4	2	7
Coronary injury (post-op)	3	0	2	2
PDA recanalization and stenting	1	1	No	No
Tunnel stenosis (ccTGA)	1	1	ECMO	1
HLHS shunt intervention	2	1	No	1
ASD device embolization	1	0	No	No

PDA, Persistent ductus arteriosus; ccTGA, Congenitally corrected transposition of the great arteries; Post-op, Postoperative.

### Off-hours catheterization-related complications

No early complications were observed. One patient, who weighed 15 kg with failing Fontan circulation, underwent LPA and azygos stenting using a 10 F sheath and developed a late femoral arteriovenous fistula, which required surgical revision after unsuccessful conservative management.

## Discussion

ECCs performed outside regular hours—during evenings, nights, and weekends—pose significant clinical and logistical challenges (5)_._ While prior pediatric studies compared urgent vs. elective catheterizations and examined procedure duration or adverse outcomes, none focused specifically on off-hours emergent interventions in CHD (1, 2). Similarly, in adult cardiology, off-hours interventions have been associated with procedural delays and, in some studies, worse outcomes. To our knowledge, this work is the first study to evaluate both outcomes and institutional facilitators for off-hours emergent catheterizations in CHD.

Compared with the large number of off-hours emergent catheterizations performed for coronary disease in adults, emergent catheterizations in CHD remain relatively uncommon, reflecting the much lower prevalence of CHD. In adult populations, outcomes have varied across studies, depending largely on institutional strategies for managing emergent cases. Some reports demonstrated worse outcomes during off-hours than during regular working hours (5, 6), whereas others found no significant differences (7–9)_._ The latter observation aligns with our findings in CHD patients and underscores the importance of a fully prepared on-call team capable of supporting a single interventionalist. Such a team includes professional anesthesiology services, specialized catheterization nursing staff, and an on-call surgical team available for immediate backup.

In our cohort, all procedures were performed by a single interventionalist with one assisting nurse under conditions of high time pressure and reduced staffing. Despite these challenges, favorable outcomes were achieved in most cases, including critically ill neonates and postoperative children with cyanosis, cardiogenic shock, or hemodynamic instability. Complication rates were minimal, and the majority of patients exhibited immediate clinical improvement. These findings support the feasibility and safety of off-hours emergency catheterization in CHD when conducted within an adequately resourced institutional framework.

### Role of non-physician and anesthesia staff

A key insight from this single-center experience is the vital contribution of non-physician personnel. In our cohort, catheterization nurses, medical technical assistants (MTAs), and operating theater assistants (OTAs) played a pivotal role in executing highly complex procedures—such as ductal recanalization, pulmonary stenosis stenting, shunt stenting, coarctation interventions, and both pulmonary and aortic valvuloplasty—often with minimal direct physician involvement. Their responsibilities extended well beyond technical support, encompassing patient monitoring, maintenance of sterile environments, and radiation safety. This reflects not only their advanced competence but also a remarkable degree of autonomy and resilience under pressure.

In this singl-center study, the anesthesiology team played a pivotal role in managing hemodynamically unstable patients, providing continuous monitoring throughout the interventions. Their expertise was crucial in maintaining stability and responding promptly to any complications that arose during procedures. Notably, in more than half of the Rashkind procedures, the on-call anesthesiologist not only accompanied the intervention but also provided real-time echocardiographic guidance—extending well beyond standard sedation duties—while simultaneously ensuring the patient's physiological stability. This broadened role was vital when ICU physician numbers were not available. In high-risk scenarios—including potential ECMO candidates—the combined expertise of nurses, MTAs, and anesthesiologists enabled the safe completion of procedures without additional physician backup.

### Institutional infrastructure

Institutional design was a major determinant of outcomes. The cardiac catheterization laboratory's location—within the surgical suite and adjacent to two operating rooms and the delivery room—enabled rapid and safe transfer of both critically ill neonates and postoperative patients, with or without ECMO support. The ICU is located adjacent to the surgical suite, allowing timely transfer of unstable patients. The full anesthesiology team was present for all emergent procedures, while the surgical team remained on call for any emergency and was present in the catheterization lab as backup before the start of emergent catheterization for all postoperative patients, as well as for those who were unstable or potential candidates for ECMO. Although not universally replicable, this structural setup demonstrates how thoughtful spatial planning and staffing architecture can significantly enhance procedural safety during emergencies.

We emphasize that all interventions were performed during evenings and nights, with a second interventionalist available on call whenever a professional nurse or fellowship assistant was unavailable.

### Broader implications

#### Three broader lessons emerge from our findings

1. Team over individual – Successful off-hours interventions depend not only on operator skill but also on coordinated, cross-trained teams, emphasizing the importance of structured simulation-based training and credentialing for all staff.
2. Timely access matters – Rapid access to catheterization may avert ECMO escalation in select patients, thereby reducing morbidity and institutional resource burden.
3. Recognition of non-physician staff – Institutional policies must acknowledge and support the indispensable role of non-physician contributors, ensuring readiness and resilience even in settings with limited physician availability.

### Limitations

This study has several limitations. First, its retrospective, single-operator, and single-center design may restrict generalizability. Second, the modest sample size reflects the inherent rarity of CHD compared with adult coronary disease; consequently, the absolute number of patients who required emergent catheterization is low, limiting statistical power. Finally, because this study did not include a direct comparison with daytime interventions, temporal differences in outcomes could not be fully assessed.

## Conclusion

Emergency congenital cardiac catheterization during off-hours is both feasible and safe when supported by a highly trained, multidisciplinary team and appropriate infrastructure. Success is not attributable to the interventionalist alone but hinges on collective expertise. Institutions should prioritize structured training, simulation readiness, and acknowledgment of non-physician staff contributions to optimize outcomes for this vulnerable population.

## Data Availability

The original contributions presented in the study are included in the article/Supplementary Material, further inquiries can be directed to the corresponding author.
